# The role of gut microbiota in Hirschsprung’s disease: from pathogenic mechanisms to microbiota-targeted therapies

**DOI:** 10.7717/peerj.20854

**Published:** 2026-02-24

**Authors:** Yuan Zhao, Dehong Chen, Kaiwen Qi

**Affiliations:** Pediatric Ward 3, Jinan Maternal and Child Care Hospital, Jinan, Shizhong District, China

**Keywords:** Hirschsprung’s disease, Intestinal microenvironment, Neuro-immune response, Probiotics, Fecal microbiota transplantation

## Abstract

Hirschsprung’s disease (HSCR) is a common congenital disorder characterized by abnormal enteric nervous system development. Recent studies have demonstrated that gut microbiota and their metabolites play a significant role in the pathogenesis of HSCR. This review systematically examines the interplay between gut dysbiosis and pathophysiological alterations in HSCR, including disruptions in microbial composition, aberrant metabolite profiles, impaired intestinal barrier function, and dysregulated neuro-immune modulation. Research indicates that HSCR patients exhibit a characteristic gut microbial imbalance, which may influence the development and function of the enteric nervous system by altering the intestinal microenvironment, including metabolic profiles and immune status. Furthermore, this review explores the potential therapeutic value of microbiota-targeted interventions, such as probiotics and fecal microbiota transplantation (FMT), in HSCR treatment, providing a theoretical foundation for novel therapeutic strategies. These findings not only enhance the understanding of HSCR pathogenesis but also offer new perspectives for clinical prevention and treatment.

## Introduction

Hirschsprung’s disease (HSCR) is one of the most common congenital intestinal anomalies in infants (Zitouni et al., 2025). The estimated incidence of HSCR in live births is approximately 1/5,000, though reported rates vary widely, ranging from 1/2,000 to 1/12,000 (Anderson et al., 2018). The disease exhibits a marked male predominance (male-to-female ratio 4:1) and is characterized by the absence of ganglion cells in the distal colon (Brits et al., 2025). This aganglionosis leads to persistent intestinal spasm, resulting in fecal accumulation in the proximal colon, which undergoes thickening and dilation under chronic fecal stimulation (Ali, Haider & Alhindi, 2021). HSCR most frequently affects the rectosigmoid region or the entire colon, presenting with typical symptoms such as constipation, progressive abdominal distension, poor feeding, failure to thrive, and functional obstruction of the distal colon (Das & Mohanty, 2017). Surgical intervention remains the primary treatment for HSCR, with various techniques including the Swenson, Duhamel, Soave (endorectal pull-through), Rehbein, Martin, and transanal endorectal pull-through procedures. The core principle involves resection of the aganglionic bowel segment and reconstruction to connect the normally innervated intestine to the anus while preserving sphincter function (Smith, Ambartsumyan & Kapur, 2020). Although most children experience significant symptomatic improvement and prolonged survival postoperatively, approximately one-third of patients develop postoperative complications (Gosain, 2016). Impaired bowel function is particularly common (Gunadi et al., 2021; Bjørnl et al., 2017), manifesting as fecal incontinence, soiling, constipation, or recurrent enterocolitis. Untreated cases often lead to mortality in childhood due to bacterial bloodstream infections secondary to intestinal inflammation (enterocolitis) or perforation (Seo et al., 2018; Askarpour et al., 2021; Jiang et al., 2025). Among these complications, Hirschsprung-associated enterocolitis (HAEC) is the most prevalent (Gosain et al., 2017), with a mortality rate of approximately 5%, making it the leading cause of death in HSCR patients (Heuckeroth, 2018). Notably, while conventional surgical treatment addresses the anatomical defect of aganglionosis, it fails to fully restore functional intestinal disorders. Therefore, there is an urgent need to explore novel therapeutic strategies beyond traditional surgery to improve intestinal function and reduce the risk of complications such as HAEC.

Currently, multiple genes have been implicated in the pathogenesis of HSCR, including *RET*, *GDNF*, *ECE1*, *EDN3*, *NRTN*, *SOX10*, *PHOX2B*, and *NRG1* (Heuckeroth, 2018; Tilghman et al., 2019; Chatterjee et al., 2021). However, the disease’s pathogenic mechanisms remain incompletely understood. Growing evidence suggests that, in addition to genetic factors, alterations in the intestinal microenvironment play a crucial role in the development of the enteric nervous system (ENS). Among these factors, the gut microbiota (GM) has emerged as a novel research focus—not only as the most complex and dynamic microbial ecosystem in the human body but also as a key regulator in maintaining homeostasis between the host and external environment. A balanced GM is essential for infant growth, development, and immune system maturation (Conroy, Shi & Walker, 2009). The human gut harbors approximately 1,000 bacterial species, totaling 1*10^14^ microorganisms, with Firmicutes and Bacteroidetes dominating (∼90% of the total GM). These commensal microbes engage in a symbiotic relationship with the host, maintaining a dynamic equilibrium of mutual dependence and regulation. Studies have identified a significant causal relationship between GM dysbiosis and HSCR, with Clostridiaceae, Ruminococcaceae, Tenericutes RF9, Peptococcus, Ruminococcus, and Paraprevotella acting as protective factors, whereas Eggerthella increases HSCR risk (Liu et al., 2024). Furthermore, Shi et al. (2024) demonstrated that Bifidobacterium, Lactobacillus, and Veillonella were significantly enriched in healthy individuals, while Enterococcus was overrepresented in those with a history of HAEC. These findings provide a novel microbiome-based perspective on HSCR pathogenesis and suggest that GM modulation may represent a promising therapeutic target for HSCR and its complications. Based on this evidence, our study systematically evaluates the potential of microbiota-targeted interventions in HSCR treatment.

This literature review is intended for a diverse audience of researchers and clinical practitioners in the fields of pediatric surgery, gastroenterology, and microbiology. It aims to provide clinical gastroenterologists and pediatric surgeons with a comprehensive understanding of the latest advancements in gut microbiota research related to Hirschsprung’s disease (HSCR), offering insights into the potential of microbiota-targeted therapies for improving postoperative outcomes. Additionally, it serves as a valuable reference for basic researchers focusing on the enteric nervous system and neuro-immunal regulation, facilitating interdisciplinary collaboration and inspiring novel investigative approaches. We anticipate that this review will not only aid clinicians in developing innovative strategies for managing HSCR and its complications but also provide researchers with a solid theoretical foundation for further exploration into the mechanisms underlying microbiota-host interactions.

### The gut microbiota

The gut microbiota (GM), as the largest microecosystem in the human body, plays a central role in maintaining intestinal homeostasis and host health (Bäckhed et al., 2015). Under normal conditions, a mutualistic symbiotic relationship exists between the host and the GM: the intestine provides a habitat for microorganisms, while the GM contributes to key physiological processes—such as intestinal barrier formation, immune regulation, and neural development—through the synthesis of bioactive compounds, including vitamins, tryptophan metabolites, and short-chain fatty acids (SCFAs). In terms of immune modulation, microbial metabolites such as SCFAs (*e.g.*, butyrate) and tryptophan enhance intestinal integrity, stimulate group 3 innate lymphoid cells (ILC3s) to produce interleukin-22 (IL-22), promote the differentiation of naïve CD8+ T cells into CD4+ T cells, regulate the expression of pro-inflammatory cytokines (*e.g.*, IL-6, IL-12, and TNF-α), and induce IgA secretion to mitigate inflammation (Mcdermott & Huffnagle, 2014; Yoo et al., 2020). Additionally, the GM strengthens intestinal barrier function by promoting mucosal defense and epithelial proliferation, thereby reducing intestinal permeability and enhancing epithelial defense mechanisms (Mcdermott & Huffnagle, 2014; Yoo et al., 2020; Shi et al., 2017). Colonization of the GM begins during the neonatal period and undergoes dynamic succession in early developmental stages. Notably, the structural and functional evolution of the GM coincides with ENS development (Frese & Mills, 2015; Gritz & Bhandari, 2015; Stewart et al., 2018). Although bacterial colonization of the intestine begins after birth, when the ENS structure is already formed, accumulating evidence indicates that the microbiota continues to influence the maturation, plasticity, and function of the ENS during the neonatal period. Early postnatal microbial exposure modulates neurogenesis, glial differentiation, and neurotransmitter expression through microbial metabolites such as SCFAs, tryptophan derivatives, and bile acids (Zhang et al., 2025; Schill, Floyd & Newberry, 2022). Moreover, maternal microbiota–derived metabolites may cross the placenta or affect fetal immune programming, indirectly shaping ENS development before birth (Vuong et al., 2020). Therefore, microbial influences on the ENS should be understood as modulatory rather than initiatory, primarily affecting postnatal maturation and functional adaptation rather than embryonic morphogenesis.

### Interplay between GM and HSCR

**Figure 1:**
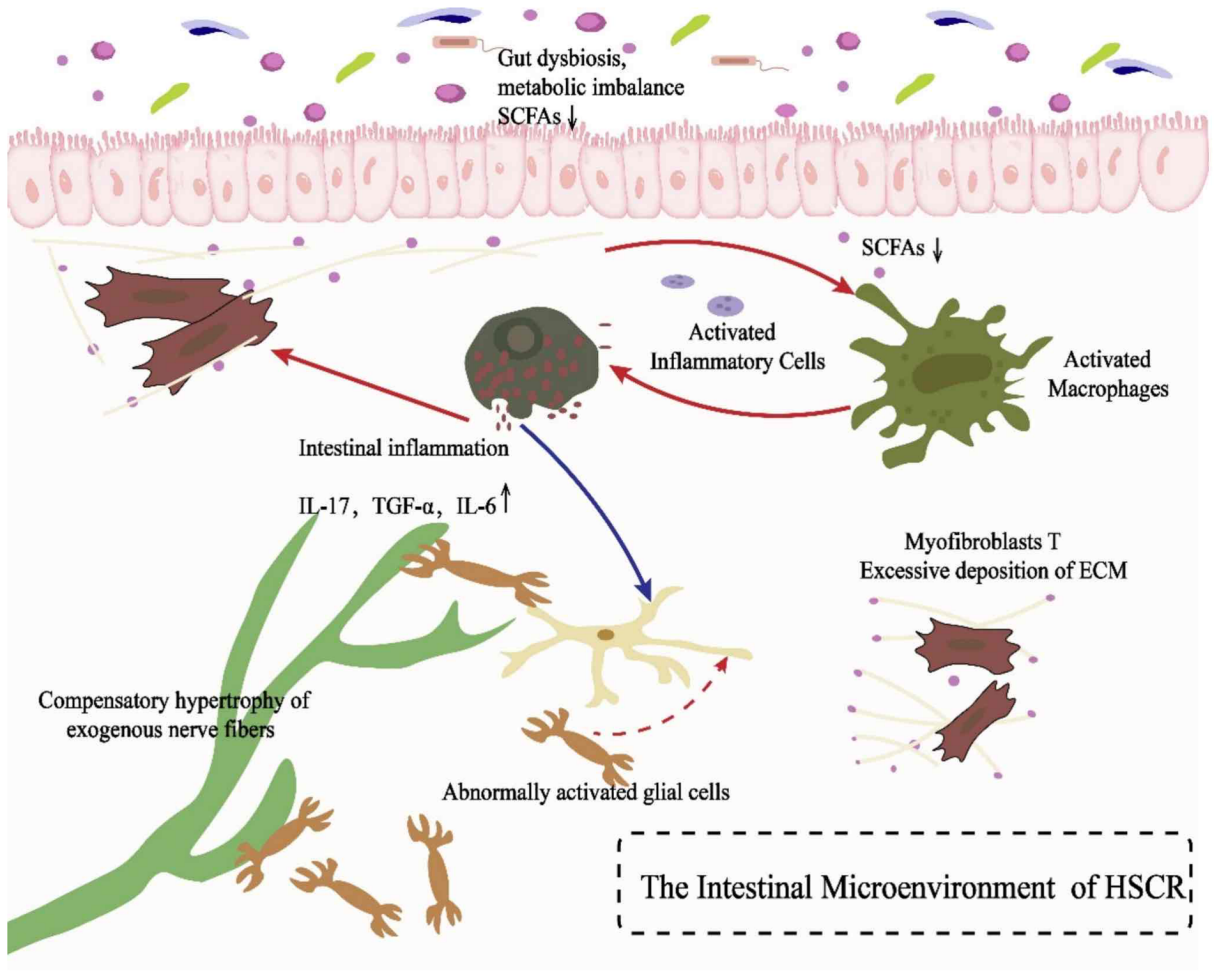
Alterations in the intestinal microenvironment of HSCR and their potential mechanisms. An illustration of the intestinal microenvironment disorder and its possible pathological mechanisms in HSCR: Gut microbiota disorder (microbiota disorder) and metabolic imbalance (metabolic imbalance) lead to decreased levels of short-chain fatty acids (SCFAs), which activate inflammatory cells (activated inflammatory cells) and macrophages (activated macrophages), triggering intestinal inflammation (intestinal inflammation). During the inflammatory response, levels of pro-inflammatory factors such as IL-17, TGF-α and IL-6 increase, further stimulating myofibroblasts (myofibroblasts) and excessive deposition of extracellular matrix (ECM). Meanwhile, compensatory hypertrophy of extrinsic nerve fibers (compensatory hypertrophy of exogenous nerve fibers) and abnormal state glial cells (abnormal state glial cells) may exacerbate dysfunction of the enteric nervous system, collectively participating in the pathological process of HSCR.

The characteristic intestinal ganglion cell deficiency in HSCR not only leads to intestinal dyskinesia, but also triggers a series of complex microenvironmental changes. Existing studies have shown that there are three key pathophysiological alterations in the pathological state of HSCR: (1) intestinal microecological dysregulation (including disturbances in the composition of the intestinal flora and metabolite abnormalities); (2) impaired function of the epithelial barrier (Pardy et al., 2021); (3) abnormalities in the neuro-immune response (Fig. 1).

#### Gut microbiota dysbiosis

Recent studies have revealed a close association between HSCR and GM dysbiosis. Pierre et al. (2014) found that α-diversity of fecal microbiota in Ednrb knockout mice continuously increased over time, with significant differences in β-diversity observed in advanced HSCR mice. Arnaud et al. (2021) reported significant differences in β-diversity between HSCR and control groups. Cheng et al. (2018) demonstrated that α-diversity in HSCR mice was higher than in wild-type mice and increased with age, but decreased after pull-through surgery. A cross-sectional study showed reduced GM richness in HSCR children after radical surgery compared to controls (Neuvonen et al., 2018). Cong et al. (2018) found significantly decreased α-diversity in post-operative HSCR patients compared to pre-operative and healthy adult controls, consistent with animal studies.

Studies have shown that in the HSCR mouse model, the intestinal flora showed significant changes at the phylum level, with a significant increase in the abundance of Proteobacteria and Bacteroidetes, and a decrease in Actinobacteria and TM7, Thick-walled Bacteria (Pierre et al., 2014; Arnaud et al., 2021; Neuvonen et al., 2018; Ward et al., 2012; Touré et al., 2019). At family and class levels, Enterobacteriaceae and Bacilli showed abnormal increases (Neuvonen et al., 2018). Genus-level analysis revealed elevated Escherichia (Proteobacteria) (Pierre et al., 2014), Bacteroides (Bacteroidetes) (Pierre et al., 2014; Ward et al., 2012), and Tannerella (Ward et al., 2012), while Lactobacillus (Pierre et al., 2014; Ward et al., 2012) and Staphylococcus (Firmicutes) (Ward et al., 2012) decreased. Notably, Arnaud et al. (2021) observed pro-inflammatory bacteria enrichment (Fusobacterium, Mogibacterium, Bilophila) in rectosigmoid regions of HSCR piglets. Clinical studies in HSCR children similarly found overgrowth of Macellibacteroides and Pseudomonas (Proteobacteria), along with increased Prevotella (Bacteroidetes) and Actinomyces (Actinobacteria) (Neuvonen et al., 2018). Proteobacteria overgrowth, a hallmark of dysbiosis, may elevate inflammation risk and impair pathogen defense (Shin, Whon & Bae, 2015). Postoperative HSCR patients showed significant reductions in Lactobacillus (Firmicutes), Lachnospiraceae, and Ruminococcaceae (Neuvonen et al., 2018; Shen et al., 2009). It is important to note that most of the above findings are based on cross-sectional or observational analyses, which establish associations rather than causal relationships between microbiota alterations and HSCR. Further mechanistic studies using germ-free or gnotobiotic models are needed to verify causality.

#### Abnormal metabolites

Short-chain fatty acids (SCFAs), including butyrate, propionate, and acetate, are key functional metabolites produced by gut microbiota through dietary fiber fermentation. These metabolites not only serve as energy sources for intestinal epithelial cells but also critically regulate ENS development and function (Dalile et al., 2019). Notably, HSCR patients exhibit significant structural and functional abnormalities in the ENS (Montalva et al., 2023). Animal studies demonstrate that SCFAs promote the differentiation of intestinal neural stem cells and effectively improve enteric nervous dysfunction (Tian et al., 2023; Wang et al., 2023). These results provide experimental evidence supporting a causal link between specific microbial metabolites and ENS development, in contrast to the largely correlational observations in human studies. Additionally, SCFAs selectively increase the proportion of excitatory cholinergic neurons in the colon, thereby modulating intestinal motility (Soret et al., 2010).

Demehri et al. (2016) found that HSCR children with HAEC history had fecal SCFA levels reduced to 1/4 of non-HAEC HSCR patients. HAEC cases showed decreased acetate but relatively increased butyrate, along with altered SCFA profiles, suggesting complex interactions between colonic metabolome changes and gut dysbiosis in HAEC pathogenesis. Liu et al. (2024) MR showed that some SCFA-producing bacteria, such as Peptococcus, Ruminalococcus 2, Clostridiaceae 1, and Ruminalococcaceae, had a protective effect against HSCR, and some of these flora were significantly reduced and correlated with the levels of SCFAs in HSCR patients Among them, rumenococci 2 and *Clostridium difficile* family 1 are involved in the metabolism of bile acids (Tanaka et al., 2020; Wang et al., 2020; Molinero et al., 2022), which activate the G protein-coupled bile acid receptor (TGR5) (Lin et al., 2023). Secondary bile acids have been shown to stimulate the secretion of 5-HT, glucagon-like peptide 1 (GLP-1) and calcitonin gene-related peptide (CGRP), all of which significantly regulate ENS and intestinal motility (Bampton et al., 2002; Kidd et al., 2008; Alemi et al., 2013). Thus, specific gut microbes (*e.g.*, Peptococcus, Ruminococcus, Clostridiaceae, Ruminococcaceae) regulate ENS maturation through bioactive SCFAs and bile acids, thereby reducing HSCR risk.

Collectively, these findings indicate that metabolic dysregulation in HSCR is closely linked to microbial alterations, with SCFAs and bile acids serving as key mediators connecting microbiota composition and ENS development. Nevertheless, metabolite profiles reported across studies remain inconsistent, suggesting that host genetic factors, dietary composition, and microbial community structure jointly shape the metabolic landscape. A more integrative metabolomics–microbiome approach will be essential to disentangle these complex interactions.

#### Disruption of intestinal barrier function

The intestinal epithelium is the largest and most important barrier against the external environment and maintains its selective barrier function through the formation of a complex protein-protein network that connects the epithelial cells to form three adhesion complexes: bridging particles, adhesion junctions, and tight junctions, which consist of transmembrane proteins. Intestinal barrier’s leads to intestinal inflammation and systemic infectious complications (Groschwitz & Hogan, 2009; Citi, 2018). It has been shown that patients with HSCR have reduced mucus turnover, decreased mucin concentration, abnormal mucin ratio (neutral mucin: acid-sulfate mucin) (Corfield et al., 2000), as well as altered size and proliferative capacity of mucus-secreting cuprocytes (Yoshimaru et al., 2024), and that passive diffusion of particulate matter and active microbial transport are compromised, as compared to the healthy state (Yildiz et al., 2015).

Aslam, Spicer & Corfield (1997) demonstrated abnormal mucin metabolism by radiolabeling techniques in both aganglionic and seemingly normal aganglionic intestinal segments in patients with HSCR. Thiagarajah et al. (2014) found altered colonic epithelial cells in both aganglionic and aganglionic segments in patients and mice with HSCR. Structurally, cuprocytes were altered with an increase in the number of cuprocytes and a decrease in intracellular mucins in biopsied distal colon of HSCR patients. MUC-2 is a major mucin expressed in humans that prevents bacterial translocation across the intestinal wall (Gork et al., 1999). In a clinical study, Mattar, Coran & Teitelbaum (2003) found that the production of MUC-2 was markedly inhibited in HSCR patients. Decreased MUC-2 protein expression can lead to decreased intestinal epithelial barrier function and induce HAEC production. However, these findings are primarily derived from histological or clinical observations, and direct mechanistic evidence connecting microbial dysbiosis to barrier dysfunction in HSCR remains limited. In addition, other studies have demonstrated altered gene expression in colonic mucosal epithelial cells of HSCR patients, including tailed homology cassette genes-1 and -2 (CDX-1 and CDX-2) that control proliferation and differentiation of intestinal mucosal cells (Lui et al., 2001), intracellular adhesion molecule-1 (ICAM-1) a cell surface adhesion glycoprotein involved in leukocyte recruitment (Kobayashi, Hirakawa & Puri, 1995), and follicle protein-1, which is involved in inflammation and intestinal epithelial barrier function of fiducial protein-1 (Nakamura et al., 2019).

In summary, HSCR patients display multifactorial impairment of intestinal barrier integrity characterized by defective mucus layer formation, tight-junction disruption, and inflammatory remodeling. While histological evidence strongly supports barrier dysfunction, direct mechanistic links between microbial dysbiosis and epithelial alterations remain to be fully established. Integrative studies combining microbiome, transcriptomic, and histopathologic analyses may clarify whether barrier disruption is a primary driver or a secondary consequence of gut dysbiosis in HSCR.

#### Imbalance in neuro-immune regulation

Neuro-immune regulation plays a crucial role in maintaining homeostasis, particularly in the regulation of intestinal inflammation through a dual mechanism: on one hand, the nervous system dynamically regulates the activity and function of immune cells by releasing neurotransmitters and neuropeptides; on the other hand, the immune system participates in neuronal damage repair, regeneration and differentiation processes by secreting cytokines and other mediators, forming a bidirectional interactive regulatory network (Godinho-Silva, Cardoso & Veiga-Fernandes, 2019). HSCR primarily originates from developmental disorders of the enteric nervous system (ENS) derived from neural crest cells (NCC) (Diposarosa et al., 2021). Scholars believe that interactions between neuro-immune pathways play a significant role in HSCR (Ji, Lai & Tou, 2023).

Research indicates that mast cells (MC) are crucial in the pathogenesis of HSCR and the repair process of the ENS (Hermanowicz et al., 2008). Kobayashi et al. (1999) demonstrated that MC may play a key role in Hirschsprung’s disease and intestinal neuronal dysplasia. The nerve growth factor (NGF) secreted by MC is not only closely related to neural hypertrophy and giant ganglion formation but may also promote abnormal proliferation of cholinergic and adrenergic nerves in HSCR and intestinal neuronal dysplasia (IND) lesions by regulating neurotransmitter systems. Rintala & Lindahl (2001) found that sodium cromoglicate (SCG), a non-absorbable MC stabilizer with no systemic side effects, can effectively treat chronic or recurrent colitis in HSCR patients. This may be related to MC remodeling neural distribution in the HSCR intestine and enhancing neuromodulation. Chen et al. (2020) showed that macrophage-related inflammation directly promotes the development of HAEC in the dilated colon. Specifically, in the inflamed proximal dilated colon, macrophage infiltration increases sharply, accompanied by the loss of the C-KIT+ phenotype in interstitial cells of Cajal (ICC). The loss of the C-KIT+ phenotype impairs the pacemaker function of ICC, leading to persistent colonic dysmotility. Furthermore, macrophages polarize into a CD45+F4/80+CD11b+CD11c–pro-inflammatory phenotype, producing large amounts of iNOS and TNF-α. TNF-α, in turn, suppresses C-KIT expression in ICC *via* the NF-*κ*B/miR-221 pathway. Additionally, studies suggest that targeted therapies against IL-8 and pIgR-mediated sIgA translocation may be effective in treating HSCR-induced inflammation (Müller et al., 2021; Medrano et al., 2019). Most of these findings describe immunological correlations observed in HSCR tissues or animal models, while the direct microbial triggers and signaling pathways require further experimental validation to establish causality. To provide a more comprehensive overview of current research, Table 1 summarizes the key characteristics and findings of representative clinical and preclinical studies investigating the association between gut microbiota and Hirschsprung’s disease.

**Table 1:** Summary of representative studies investigating gut microbiota and Hirschsprung’s disease (HSCR).

Authors (year)	Study type	Model/population	Sample type	Main findings	Pathophysiological implications methodological quality/notes
Pierre et al. (2014)	Preclinical (Ednrb−/− mouse)	Mouse model	Fecal samples	Dysbiosis with ↑Proteobacteria, ↓Lactobacillus; bacterial translocation observed	Supports link between dysbiosis and inflammation in HSCR	Controlled animal experiment
Neuvonen et al. (2018)	Clinical (cross-sectional)	42 HSCR children *vs* 37 controls	Stool samples	Reduced α-diversity and beneficial genera; ↑Enterobacteriaceae	Postoperative microbiota imbalance linked to HAEC risk	Observational study
Arnaud et al. (2021)	Preclinical (piglet model)	Iatrogenic rectosigmoid hypoganglionosis	Mucosal and fecal samples	Altered β-diversity; proinflammatory taxa enriched	ENS absence disrupts mucosal barrier and microbiota maturation	Translationally relevant model
Shi et al. (2024)	Clinical	HSCR with *vs.* without HAEC	16S rRNA sequencing	↓Bifidobacterium, ↑Enterococcus in HAEC	Specific taxa linked to postoperative inflammation	Moderate sample size
Tian et al. (2023)	Preclinical	Rat hypoganglionosis + FMT	Fecal transplant	FMT restored SCFAs, promoted neural crest cell migration via MEK1/2 pathway	Demonstrates causal role of microbial metabolites	Mechanistic experiment
Liu et al. (2024)	Mendelian Randomization	Human population-level data	Genomic/GWAS	SCFA-producing bacteria (Peptococcus, Ruminococcus) protective	Suggests potential causal microbial influence	High-quality causal inference
Wang et al. (2015)	Clinical RCT	Postoperative HSCR children	Fecal samples + cytokines	Probiotics reduced HAEC incidence and TNF-α/IFN-γ	Microbiota-targeted therapy improves immune balance	Randomized controlled trial

**Notes.**

Abbreviations HAEC Hirschsprung-associated enterocolitis FMT fecal microbiota transplantation SCFA short-chain fatty acids ENS enteric nervous system

Taken together, these studies highlight the critical role of neuro-immune crosstalk in shaping the inflammatory and motility disturbances observed in HSCR. The reciprocal regulation between immune cell activation and neuronal plasticity suggests that microbial dysbiosis may act as an upstream modulator of this axis. Future research should focus on identifying specific microbial or metabolite signals that influence immune–neuronal communication and testing whether targeted modulation can restore ENS function and reduce HAEC risk.

### Impact of targeted GM on HSCR

Clinical data showed (Xie et al., 2022) that the diversity of intestinal flora in children with HAEC was significantly lower than that in children with HSCR without HAEC, characterized by (1) increased abundance of Aspergillus spp. (45.3% *vs.* 31.3%) (*P* = 0.76); (2) significantly decreased abundance of Actinobacteria spp. (8.7% *vs.* 14.1%, *P* = 0.047); and (3) the conditionally pathogenic bacteria *Klebsiella* spp. abnormally proliferated (37.5% *vs* 0.2%, *P* = 0.013). This flora disturbance may lead to impaired intestinal mucosal barrier function, thus promoting the development of HAEC.

In terms of therapeutic strategies, targeted GM shows promising applications. Probiotic intervention may protect GM homeostasis through multiple mechanisms: (1) maintaining tight junction protein expression; (2) inhibiting apoptosis in intestinal epithelial cells; (3) decreasing pathogenic bacterial colonization; (4) regulating inflammatory factor secretion (decreasing TNF-α, IFN-γ, IL-6, and elevating IL-10); and (5) increasing mucus production and secretion (Ji et al., 2023; Wan et al., 2016). Xie et al. (2022) found that probiotic intake may reduce the incidence of HAEC. A prospective multicenter randomized controlled trial by Wang et al. (2015) further demonstrated that probiotics not only significantly reduced the incidence of HAEC, but also reduced pro-inflammatory cytokines (TNF-α, IFN-γ, IL-6) and increased anti-inflammatory cytokines (IL-10) to balance the T-lymphocyte subpopulations. Mei et al. (2022) showed that enterally administered probiotics act through multiple mechanisms to influence factors associated with HAEC development, including dysregulation of GM ecology, impaired mucosal barrier function, altered innate immune response, and bacterial translocation. Moreover, in addition to probiotics, fecal microtransplantation (FMT) has shown unique therapeutic value. Animal experiments have shown that FMT can effectively increase the abundance of beneficial bacteria such as *Mycobacterium anisopliae* and *Clostridium difficile*, elevate the level of short-chain fatty acids, which in turn promotes the proliferation and migration of enteric neural crest cells through the MEK1/2-MAPK signaling pathway, and ameliorate the ganglionic hypofunction (Tian et al., 2023). These findings provide a new theoretical basis for the microecological treatment of HSCR and its complications.

### Discussion

HSCR is a congenital intestinal malformation characterized by the absence of distal colonic ganglion cells (Soares de Oliveira et al., 2021), and its pathogenesis involves a combination of genetic factors (*e.g.*, mutations in genes such as *RET*, *EDNRB*, *etc.*) and microenvironmental abnormalities (Amiel et al., 2008; Lake & Heuckeroth, 2013; Yang et al., 2017). Although surgical resection of ganglion-free intestinal segments is currently the mainstay of treatment, postoperative patients still often face complications such as intestinal dysfunction and recurrent HAEC. Cell transplantation is considered a promising strategy for radical HSCR, but faces many challenges (Yu et al., 2025). In recent years, there has been increasing evidence that GM and its metabolites play a key role in the pathogenesis and development of complications of HSCR by regulating ENS development, maintaining mucosal barrier function, and modulating immune homeostasis. Nevertheless, it should be emphasized that the majority of current clinical findings describing microbiota alterations in HSCR are correlational. Only a few experimental studies—particularly those employing germ-free or knockout animal models—have provided causal evidence linking specific microbial metabolites (*e.g.*, SCFAs, bile acids) to ENS development and intestinal function. For example, both HSCR patients and animal models exhibit characteristic dysbiosis (*e.g.*, an increase in Aspergillus phylum and a decrease in short-chain fatty acid-producing bacteria) and metabolic disturbances (*e.g.*, a decrease in butyrate levels), and these alterations may exacerbate ENS developmental deficits and postoperative intestinal dysfunction by affecting neural crest cell migration, intestinal epithelial barrier integrity, and local immune responses. An important question that remains unresolved is whether gut dysbiosis acts as a causal driver of HSCR pathogenesis or arises secondarily from the aganglionic and inflammatory intestinal microenvironment. Current evidence suggests a bidirectional relationship: on one hand, microbial imbalance and altered metabolites—such as reduced SCFAs or disrupted bile acid signaling—may impair enteric neural crest cell migration and differentiation, thereby contributing to disease onset. On the other hand, intestinal stasis, mucosal inflammation, and impaired barrier function caused by aganglionosis can themselves promote dysbiosis. This “cause–effect loop” complicates the interpretation of microbiome alterations in HSCR. While animal models and Mendelian randomization studies have provided initial causal clues linking specific taxa or metabolites to ENS development, robust longitudinal human data are still lacking.

Future research should aim to bridge this gap by integrating observational data with mechanistic experiments, which will allow a more robust understanding of the causal pathways underlying the microbiota–ENS axis in HSCR. Therefore, targeted modulation of intestinal microecology (*e.g.*, probiotics, fecal transplants, or specific metabolite interventions) may be a potential strategy to improve the prognosis of patients with HSCR and provide a theoretical rationale for new approaches beyond traditional surgical treatment.

Despite the valuable insights gained from preclinical models, several limitations should be acknowledged when extrapolating these findings to human HSCR. Most current animal models—including Ednrb-/- mice and surgically induced hypoganglionosis or rectosigmoid models—partially replicate the aganglionosis and dysbiosis seen in patients but do not fully capture the complex genetic, developmental, and environmental factors underlying human disease. Moreover, differences in gut microbiota composition, intestinal immune maturation, and neural crest cell migration between species may lead to variable responses to microbiota-targeted interventions such as probiotics or fecal microbiota transplantation. Therefore, caution should be exercised when interpreting animal data, and clinical translation requires further validation through well-designed human studies.

Building upon these limitations and mechanistic uncertainties, several key areas warrant further exploration. Future research could start from the following directions: First, the identification of reliable microbial or metabolite biomarkers could facilitate early diagnosis and risk stratification, distinguishing patients at higher risk for postoperative complications such as HAEC. Second, elucidating the mechanistic role of specific metabolites—such as SCFAs, tryptophan derivatives, and bile acids—in modulating enteric neurogenesis and immune signaling will be essential for establishing causality. Third, future therapeutic approaches should move toward combined or “multi-target” strategies that simultaneously restore microbial homeostasis, modulate immune responses, and promote enteric neuronal repair. Finally, longitudinal and interventional studies integrating microbiome, metabolomic, and clinical data will be key to translating experimental insights into precision therapies for HSCR.

## Survey Methodology

To ensure a comprehensive and unbiased coverage of the literature regarding the role of gut microbiota in Hirschsprung’s disease (HSCR), a systematic approach was employed for the identification, selection, and synthesis of relevant studies.

### Literature search strategy

We conducted electronic searches in the following databases from inception to August 2025: PubMed/MEDLINE, Web of Science, Embase, Cochrane Library, China National Knowledge, Infrastructure (CNKI), Wanfang Data. In total, 152 potentially relevant records were identified through database searching. After screening titles and abstracts, 47 records were excluded due to duplication, non-English/Chinese language, or lack of relevance to gut microbiota and HSCR. Full-text assessment of the remaining 105 studies yielded 72 original articles that met all inclusion criteria. Among these, 36 were clinical studies (including case–control or cohort designs), 22 were preclinical animal studies, and 14 were mechanistic *in vitro* investigations. In addition, 18 review articles and four meta-analyses were retrieved to provide broader contextual understanding. These secondary sources were not directly cited as evidence for specific findings, but were used to identify relevant primary studies and to place the current synthesis in the context of existing knowledge. All original articles referenced within those reviews were independently screened and re-evaluated according to the same inclusion and exclusion criteria applied in this work.

### Search terms

The search strategy combined keywords and Medical Subject Headings (MeSH) terms related to Hirschsprung’s disease and gut microbiota, including: “Hirschsprung disease” OR “HSCR”, “gut microbiota” OR “intestinal microbiome” OR “microbiome” OR “dysbiosis”, “enteric nervous system”, “short-chain fatty acids” OR “SCFA”, “bile acids”, “intestinal barrier” OR “mucin” OR “MUC-2”, “neuro-immune”, “probiotics” OR “fecal microbiota transplantation” OR “FMT”, “Hirschsprung-associated enterocolitis” OR “HAEC”. Boolean operators (AND, OR) were used to combine terms. The search was not restricted by language initially, but the final synthesis focused on articles published in English and Chinese.

### Inclusion and exclusion criteria

#### Inclusion

Original research articles (both human and animal studies), reviews, meta-analyses, and clinical trials focusing on gut microbiota composition, microbial metabolites (*e.g.*, SCFAs, bile acids), intestinal barrier function, neuro-immune interactions, and microbiota-targeted therapies (*e.g.*, probiotics, FMT) in the context of HSCR.

#### Exclusion

Studies not related to HSCR, editorials, letters, conference abstracts without full text, and studies lacking primary data or relevant outcomes.

### Study selection and data extraction

Two reviewers independently screened titles and abstracts for eligibility. Full-text articles of potentially relevant studies were assessed based on the inclusion criteria. Any discrepancies were resolved through discussion or consultation with a third reviewer. Data extracted included author, publication year, study design, sample characteristics, key methodological details, and findings relevant to the review’s focus.

### Quality assessment

The quality of included studies was assessed using appropriate tools, such as the Newcastle-Ottawa Scale for observational studies and the Cochrane Risk of Bias Tool for randomized controlled trials.

### Synthesis

A narrative synthesis was performed to summarize and critically evaluate the evidence regarding gut microbiota dysbiosis, metabolic alterations, barrier dysfunction, neuro-immune dysregulation, and the potential of microbiota-targeted therapies in HSCR. This methodological approach ensured a rigorous, reproducible, and unbiased review of the current evidence linking gut microbiota to the pathogenesis and potential treatment of Hirschsprung’s disease.

## Conclusions

In summary, current evidence supports a close association between gut dysbiosis and HSCR pathophysiology, although causality remains to be fully established. Current management of HSCR remains challenging. Although surgical resection of aganglionic bowel segments alleviates anatomical obstruction, it frequently leads to persistent intestinal dysfunction and high incidence of HAEC. Although current evidence strongly supports an association between gut dysbiosis and HSCR pathogenesis, most data remain observational. Establishing causality will require integrative mechanistic and interventional studies in both animal models and human cohorts.

##  Supplemental Information

10.7717/peerj.20854/supp-1 Supplemental Information 1 PRISMA checklist

10.7717/peerj.20854/supp-2 Supplemental Information 2 PRISMA flowchart

## Additional Information and Declarations

### Data Availability

The following information was supplied regarding data availability: This is a literature review.
