# Supplementary figures and images for: The role of gut microbiota in Hirschsprung’s disease: from pathogenic mechanisms to microbiota-targeted therapies
Source: PeerJ. 2026 Feb 24;14:e20854. doi: 10.7717/peerj.20854 (PMC12947761; doi:10.7717/peerj.20854)

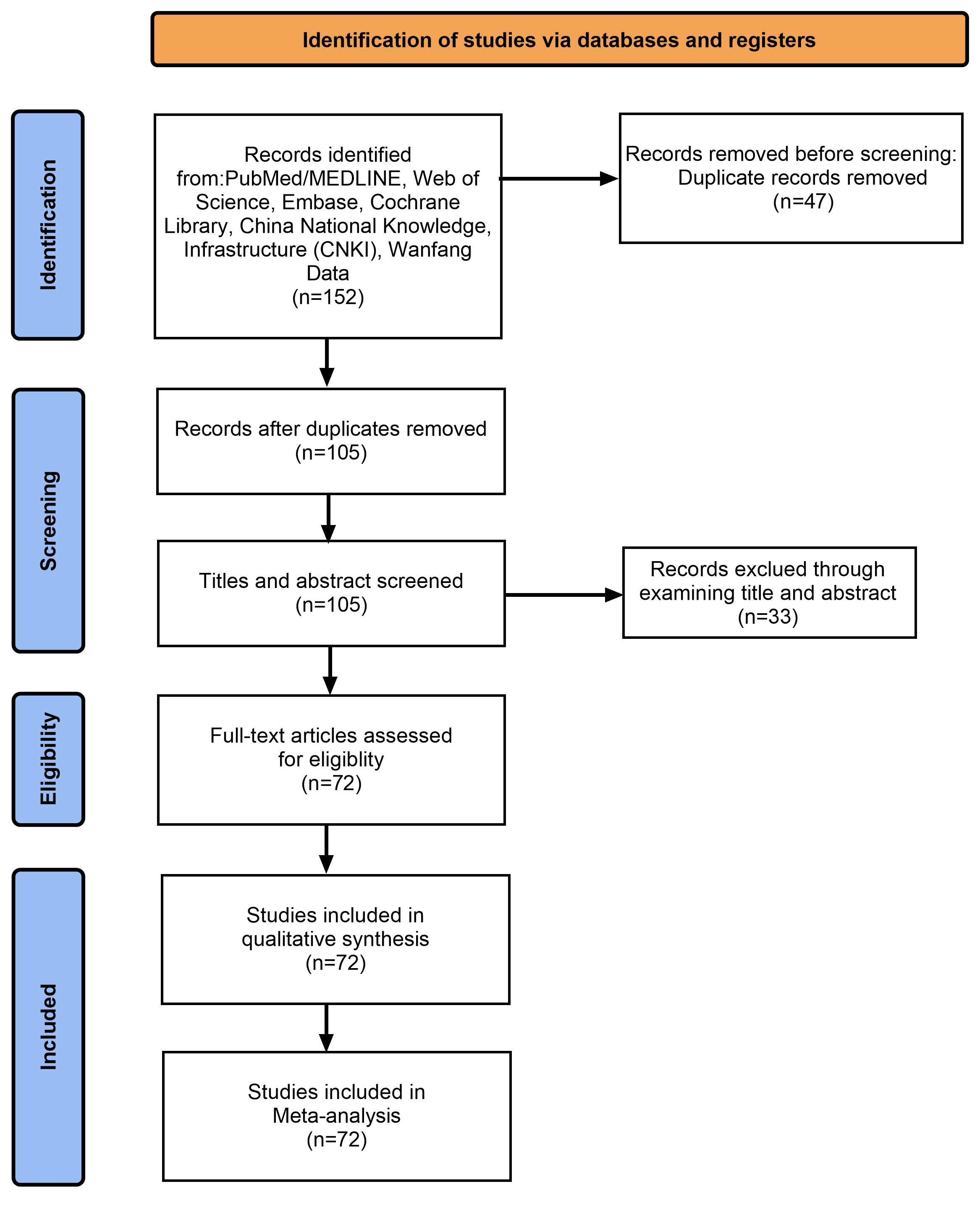

Supplement: Supplemental Information 2 [file peerj-14-20854-s002.tif]
